# Supplementary material for: A Complex Genomic Rearrangement Resulting in Loss of Function of SCN1A and SCN2A in a Patient with Severe Developmental and Epileptic Encephalopathy
Source: Int J Mol Sci. 2022 Oct 26;23(21):12900. doi: 10.3390/ijms232112900 (PMC9656590; doi:10.3390/ijms232112900)
Supplement: Supplementary file 1 [file ijms-23-12900-s001.zip › ijms-1922355-supplementary.pdf]

International Journal of
Molecular Sciences

MDPI

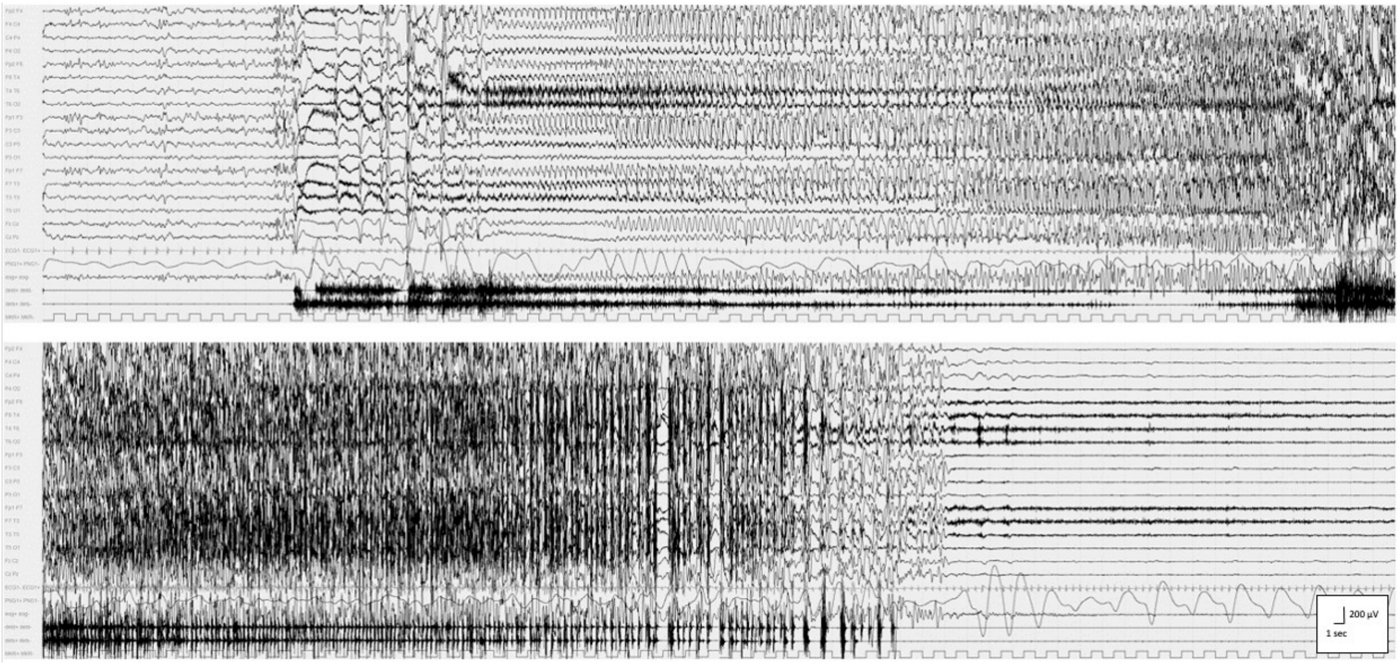

Figure S1. Ictal video-polygraphic recording of patient at the age of 5 years. The ictal discharge starts with a diffuse low-voltage fast activity, increasing in amplitude and decreasing in frequency, bilateral and symmetrical. Seizure ends after 90 seconds with intravenous midazolam administration and is followed by a post-ictal suppression of brain electrical activity (Post-Ictal Generalized EEG, PGES). Clinically: abrupt and massive tonic contraction with flushing of the face and trunk, followed by bilateral tonic-clonic contraction of upper and lower limbs 50 seconds after its beginning.
